# Relative Bioavailability Studies With Mitapivat: Formulation and Food Effect Assessments in Healthy Subjects

**DOI:** 10.1002/cpdd.1481

**Published:** 2024-10-25

**Authors:** Varsha Iyer, Karen Sullivan, Yan Yan, Peter Hawkins

**Affiliations:** ^1^ Agios Pharmaceuticals, Inc. Cambridge MA USA; ^2^ FogPharma Cambridge MA USA; ^3^ Deciphera Pharmaceuticals, Inc. (DCPH) Waltham MA USA

**Keywords:** bioavailability, biopharmaceutics, clinical pharmacology, clinical trials, drug‐food interactions, mitapivat, pharmacokinetics, pediatric formulations, rare diseases

## Abstract

Pyruvate kinase (PK) deficiency is a rare, hereditary, hemolytic anemia caused by mutations in the *PKLR* gene encoding the PK enzyme. Mitapivat (previously designated AG‐348) is a first‐in‐class, oral, allosteric activator of PK. We report results from 5 Phase 1 trials in healthy adults to characterize and compare mitapivat pharmacokinetics across different formulations and analyze food effects on mitapivat bioavailability (Studies 1‐5). Pharmacokinetic assessments were peak exposure, total exposure, time to maximum plasma concentration of mitapivat, and relative bioavailability (where appropriate). Plasma total exposure of mitapivat was similar in the fasted and fed (high‐fat meal or different soft foods) states after capsule, tablet, and pediatric granule formulations. Although mitapivat administration with food reduced the rate of mitapivat absorption (delay in time to maximum plasma concentration; reduction in maximum concentration) versus dosing under fasted conditions, this was not considered clinically relevant, given the lack of effect on total mitapivat exposure. Consequently, the administration instructions for mitapivat relating to food state that “patients may take mitapivat tablets with or without food.” These findings will continue to inform clinical studies and development of mitapivat in adult and pediatric patients with hemolytic anemias and may help inform healthcare professionals on mitapivat dosing/administration recommendations in clinical practice.

Pyruvate kinase (PK) deficiency is an underrecognized, rare, hereditary, hemolytic anemia caused by mutations in the *PKLR* gene encoding the PK enzyme.^1^ As this enzyme controls the final step of glycolysis, converting phosphoenolpyruvate to pyruvate, which results in the formation of adenosine triphosphate from adenosine diphosphate, it is critical for maintaining red blood cell (RBC) energy levels and morphology.^1^, ^2^ PK deficiency is associated with reduced RBC survival and impaired red cell maturation.^3^ This results in chronic hemolysis and leads not only to the acute and long‐term disease complications of anemia, hyperbilirubinemia, and iron overload but also to a spectrum of signs and symptoms, including jaundice and fatigue.^4^, ^5^

Mitapivat (previously designated AG‐348) is a first‐in‐class, oral allosteric activator of PK.^6^, ^7^, ^8^ Mitapivat increases the activity and stability of PK, including the wild‐type form of the enzyme and a range of mutant forms of PK found in RBCs.^7^, ^9^ In studies investigating its mechanism of action, mitapivat was shown to increase adenosine triphosphate levels and reduce levels of phosphoenolpyruvate and 2,3‐diphosphoglyceric acid, consistent with pharmacologic activation of PK enzyme activity in RBCs from patients with PK deficiency treated ex vivo.^7^

Accordingly, mitapivat has been studied for the treatment of hemolytic anemias such as PK deficiency, thalassemia, and sickle cell disease.^10^, ^11^, ^12^, ^13^, ^14^ Based on pharmacokinetic and safety data from Phase 1 studies, mitapivat was determined to have an acceptable absorption, distribution, metabolism, excretion, and safety profile for clinical testing.^8^, ^15^ Subsequent Phase 3 studies demonstrated that mitapivat was efficacious and well tolerated in adult patients with PK deficiency,^11^, ^12^ and mitapivat is now used clinically to treat hemolytic anemia in adult patients with this disease.^16^, ^17^, ^18^, ^19^

The objectives of the current study were to characterize and compare the pharmacokinetics of mitapivat among different formulations in healthy adult subjects prior to their use in patient studies and to analyze the effect of food on the bioavailability of mitapivat administered in the different formulations and doses of the drug studied in 5 Phase 1 trials. Findings from these studies are continuing to inform additional clinical studies of mitapivat in adult and pediatric patients with hemolytic anemias.

## Methods

The 5 studies included in this combined analysis were conducted in accordance with the Declaration of Helsinki, and the protocols were approved by an institutional review board prior to the start of each trial. All healthy adults who participated in the trials included in the current study provided written informed consent. The list of institutional review boards and the location of each study site are included in Table S1.

The 5 Phase 1 studies are described below. All studies were conducted in healthy subjects and assessed the following pharmacokinetic measures: peak exposure (maximum concentration [C_max_]), total exposure (area under the plasma concentration‐time curve [AUC]), time to maximum plasma concentration (t_max_) of mitapivat, and relative bioavailability (F_rel,_ calculated as the AUC from time 0 extrapolated to infinity [AUC_0‐∞_]: AUC_0‐∞_ [fed]/AUC_0‐∞_ [fasted]), where appropriate. The AUC from time 0 to the time of the last quantifiable concentration (AUC_0‐t_), calculated using the linear trapezoidal rule, and AUC_0‐∞_ were also assessed.

The study design and objectives of the 5 Phase 1 studies in healthy subjects included in this analysis are summarized in Table 1. Key subject eligibility criteria for all the studies are summarized in Table S2.

**Table 1:** Summary of Study Designs and Objectives Across the 5 Phase 1 Studies

Study	Study design	Mitapivat formulation (dose^a^) and food status	Study objectives
Study 1 (NCT02108106^8^)	Phase 1, single‐center, randomized, double‐blind, placebo‐controlled study	Capsule formulation (700 mg) Administered fasted and fed (high‐fat meal)	Single‐ascending‐dose escalation evaluation to assess the safety, pharmacokinetics, and pharmacodynamics of mitapivat capsules The effect of food on the relative bioavailability of mitapivat was evaluated in a subset of subjects
Study 2 (NCT03397329)	Phase 1, randomized, open‐label, 2‐period crossover study	Tablet formulation (1 × 50 mg) and capsule formulation (2 × 25 mg) Administered under fasted conditions	Comparison of the relative bioavailability and safety of mitapivat tablet and capsule formulations
Study 3 (NCT04472832)	Phase 1, randomized, single‐dose, double‐blinded, 4‐period crossover study	Tablet formulation (100 mg) Administered under fasted and fed (high‐fat/high‐calorie meal) conditions	Pharmacokinetics, safety, and tolerability assessment of mitapivat under fasted and high‐fat (fat representing ≈50% of the total caloric content of the meal)/high‐calorie (≈800‐1000 calories) conditions
Study 4 (NCT04696393)	Phase 1, single‐dose, open‐label, randomized, 2‐period, 2‐sequence crossover study	Tablet formulation (1 × 100 mg and 2 × 50 mg) Administered under fasted conditions	Comparison of the pharmacokinetics and safety of mitapivat 100 mg (as 1 × 100 mg or 2 × 50‐mg tablets)
Study 5 (NCT04565678)	Phase 1, single‐center, open‐label, randomized, 4‐period, 4‐sequence crossover study Subjects were randomly assigned to 1 of 4 treatment sequences	Coated granule (pediatric formulation; 50 × 1 mg) and tablet (adult formulation; 50 mg) under fasted conditions Coated granule formulation (50 × 1 mg) administered under fed (soft food [strawberry yogurt or chocolate pudding]) conditions	Comparison of the relative bioavailability of the mitapivat‐coated granule (pediatric) formulation and the tablet (adult) formulation Assessment of the effect of soft foods on the bioavailability of mitapivat administered as a coated granule formulation

a Single oral dose.

### Study 1: Effect of Food on Relative Bioavailability of a Single Ascending Dose of Mitapivat Capsule

The study design of this single‐ascending‐dose study assessing the safety and tolerability of mitapivat administered in capsule formulation under fasted conditions has been published previously by Yang et al,^8^ referred to as Period 1 (Figure S1A). Details of the internal standards used, method of extraction, chromatographic separation and mobile phase, mass spectrometer instrument, mass transitions monitored, and limits of sensitivity were previously described.^8^ The study also examined the effect of food on the bioavailability of mitapivat, involving 1 cohort with a fixed sequence design, in which subjects returned for subsequent dosing with mitapivat 700 mg under fed conditions, referred to as Period 2 (Figure S1A). During this study, mitapivat and placebo were administered orally in Swedish orange opaque size 0 gelatin capsules. Capsules were hand‐filled and prepared by an on‐site unblinded pharmacist.

#### Analysis

The pharmacokinetic population consisted of all subjects who received at least 1 dose of mitapivat and had evaluable pharmacokinetic data. The safety population consisted of all subjects who received at least 1 dose of mitapivat or placebo. The pharmacokinetic analysis was performed by PharSight Consulting Services. Geometric means and coefficients of variation were analyzed for appropriate pharmacokinetic and/or pharmacodynamic parameters. No food effect was concluded if the 90% confidence intervals (CIs) for the ratios of geometric means were contained within the interval of 80%‐125% for the C_max,_ AUC_0‐t_, and AUC_0‐∞_ of mitapivat for fasted versus fed subjects.

### Study 2: Relative Bioavailability and Safety of Mitapivat Tablet and Capsule Formulations

This study compared the F_rel_ and safety of the mitapivat tablet and capsule formulations under fasted conditions (Figure S1B). For the 2 treatment periods, plasma samples for pharmacokinetic analysis were collected before the dose of mitapivat and at time intervals up to 72 hours after dosing.

#### Analysis

The pharmacokinetic population consisted of all subjects who received at least 1 dose of mitapivat (regardless of formulation) and had evaluable pharmacokinetic data. The safety population consisted of all subjects who received at least 1 dose of mitapivat. The pharmacokinetic analysis was performed by Covance Early Clinical Biometrics using Phoenix WinNonlin Version 6.4 (PharSight Corporation). Pharmacokinetic parameters were determined for each subject on the basis of the plasma concentrations of mitapivat and using noncompartmental methods. Geometric least squares (LS) means and coefficients of variation were analyzed for appropriate pharmacokinetic and/or pharmacodynamic parameters. No difference between the tablet and capsule formulations was concluded if the 90% CIs for the ratios of geometric means were contained within the interval of 80%‐125% for C_max,_ AUC_0‐t_, and AUC_0‐∞_ of mitapivat.

### Study 3: Safety and Tolerability of Mitapivat Under Fasted and High‐Fat Conditions

In this study of mitapivat under fasted and high‐fat meal conditions, a Williams design‐based sequence was generated for randomization, and subjects were randomly assigned to 1 of 4 treatment sequences on Day 1 prior to the first dose (Figure S1C). For the purposes of this study, only the mitapivat treatments (mitapivat 100 mg [2 × 50 mg] tablets administered with 4 matching placebo tablets) either under fasted conditions or following a high‐fat breakfast were considered; neither the placebo‐only treatment (6 matching placebo tablets) nor the mitapivat 300 mg (6 × 50 mg) tablets under fasted conditions were considered.

During the study, water was permitted as desired except 1 hour before and 1 hour after administration of the study drug. For the high‐fat and high‐calorie breakfast, fat accounted for approximately 50% of the total caloric content of the meal and the meal was approximately 800‐1000 calories. During nonfasting periods, subjects received standardized meals scheduled at the same time in each period of the study.

#### Analysis

The pharmacokinetic population consisted of all subjects who received at least 1 dose of mitapivat and had evaluable pharmacokinetic data. The safety population consisted of all subjects who received at least 1 dose of mitapivat or placebo. All statistical analyses were performed using SAS Version 9.4 or higher (SAS Institute). During each treatment sequence, serial blood samples were collected before dosing and at time intervals up to 120 hours after dosing for the measurement of mitapivat concentrations. A pharmacokinetic analysis was performed using mitapivat concentrations versus time data. A statistical comparison of the mitapivat‐fed versus mitapivat‐fasted populations was performed. No food effect was concluded if the 90% CIs for the ratios of geometric means were contained within the interval of 80%‐125% for the C_max,_ AUC_0‐t_, and AUC_0‐∞_ of mitapivat from the fed and fasted conditions.

### Study 4: Pharmacokinetics and Safety of Mitapivat Administered as 100 mg or 2 × 50 mg Tablets

This study assessing the pharmacokinetics and safety of mitapivat 100‐mg tablets under fasted conditions consisted of 2 treatment periods, incorporating a washout period and a follow‐up telephone call for safety assessment (Figure S1D). In each treatment period, serial blood samples for the pharmacokinetic analysis of mitapivat were collected before dosing and up to 120 hours after dosing.

#### Analysis

The pharmacokinetic population included subjects who received at least 1 dose of mitapivat and had evaluable pharmacokinetic data. The safety population consisted of all subjects who received at least 1 dose of mitapivat. All pharmacokinetic parameters were calculated using Phoenix WinNonlin Version 8.0.0.3176 (Certara USA Inc.), or SAS Version 9.4 (SAS Institute). Blood samples were taken from each subject before dosing and at time intervals up to 120 hours after dosing to determine the plasma concentration of mitapivat. The treatments were considered similar if the 90% CI for the ratio of geometric LS means comparing the formulations (1 × 100 mg tablet vs 2 × 50 mg tablets) was completely contained within the predefined interval of 80%‐125% for all selected pharmacokinetic parameters.

### Study 5: Relative Bioavailability of Mitapivat‐Coated Granule (Pediatric) Formulation and Tablet (Adult) Formulation

This study evaluated a coated granule formulation (pediatric formulation) of mitapivat. Subjects were randomly assigned to 1 of 4 treatment sequences, each sequence having 4 periods (Figure S1E). Soft foods of different pH levels were selected to assess the effect of pH (strawberry yogurt [pH, 4.0‐5.0] and chocolate pudding [pH, 5.5‐6.5]), given that mitapivat has relatively high solubility at a pH ranging from 1.0 to 5.5 and low solubility at a pH ranging from 5.5 to 8.0.

#### Analysis

The pharmacokinetic population included all patients who received at least 1 dose of mitapivat and had evaluable pharmacokinetic data. The safety population consisted of all subjects who received at least 1 dose of mitapivat. Blood sampling to determine mitapivat concentrations in plasma took place during treatment periods 1‐4 on Day 1 (before dosing and at time intervals up to 72 hours after dosing). Plasma concentrations of mitapivat were determined using a validated liquid chromatography‐tandem mass spectrometry assay. The pharmacokinetic parameters were determined from the plasma concentrations of mitapivat using noncompartmental methods in a validated software program: Phoenix WinNonlin Version 8.1 (Certara). The treatments (fasted‐coated granule vs fasted‐tablet) were considered similar if the 90% CI for the ratio of geometric LS means was completely contained within the predefined interval of 80%‐125% for all selected pharmacokinetic parameters. The same comparison was used to determine the impact of soft food/pH.

## Results

### Pharmacokinetic Analysis

#### Study 1

**Figure 1:**
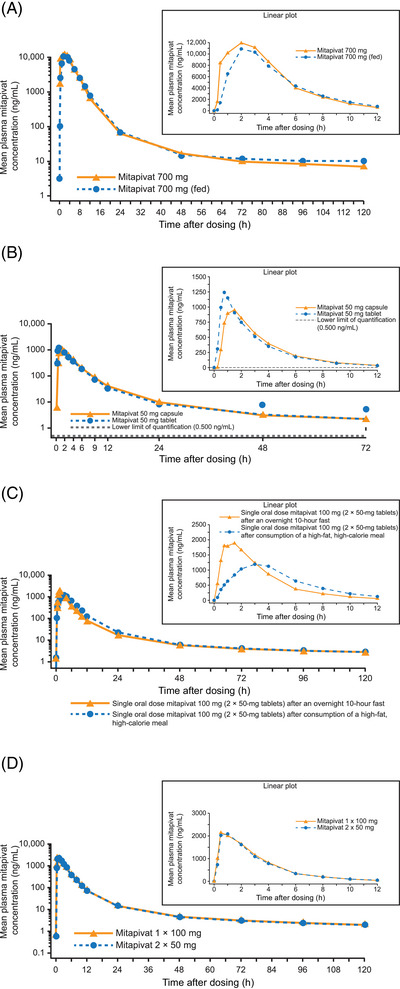
(A) Mean plasma concentrations of mitapivat versus time after single‐dose oral administration of 700 mg of mitapivat under fasting or fed conditions (Study 1). (B) Mean plasma concentrations of mitapivat following single‐dose administration of 1 × 50‐mg tablet and 2 × 25‐mg capsules (Study 2). (C) Mean plasma concentrations of 100 mg of mitapivat versus time in fasted versus fed (high‐fat, high‐calorie meal) conditions (Study 3). (D) Mean plasma concentration versus time profiles following single oral administration of 1 × 100 mg and 2 × 50‐mg mitapivat (Study 4).

Six subjects participated in the food‐effect part of the study and received a second dose of 700 mg mitapivat (n = 5, all men; mean age, 42.3 years) or placebo (n = 1, man, 42 years) under fed conditions. The mean body mass index (BMI) of these 6 subjects was 27.5 kg/m^2^ (range, 23.3‐30.2 kg/m^2^). Mean plasma concentration‐time profiles of mitapivat following single‐dose oral administrations of the 700‐mg dose under both fasting and fed conditions are shown in Figure 1A, and pharmacokinetic parameters are summarized for all studies in Tables 2 and 3. In this study, absorption was rapid, and exposure to mitapivat, as measured by AUC and C_max_, was similar following a single 700‐mg dose of mitapivat in both fasted and fed conditions (C_max_, 12,686  and 11,304 ng/mL, respectively; Table 2). In addition, the mean AUC_0‐t_ and AUC_0‐∞_ were comparable under fasted and fed conditions (Table 2). There were also no significant differences in median t_max_ under both fasted and fed conditions (1.49 and 2.05 hours, respectively; Table 2).

**Table 2:** Plasma Pharmacokinetic Parameters of Mitapivat across Studies by Dose, Formulation, and Fed or Fasted Status

Study, N	Dose (mg)	Mitapivat formulation	Fed/fasted status	t_max_ (hour), median (range)	C_max_ (ng/mL)	AUC_0‐t_ (ng.h/mL)	AUC_0‐∞_ (ng•h/mL)	F_rel_	C_max_ (ng/mL)	AUC_0‐t_ (ng•h/mL)	AUC_0‐∞_ (ng•h/mL)	t½ (hour)	F_rel_
					Geometric mean (geometric CV%)				Arithmetic mean (SD)				
Study 1													
n = 5	700	Capsule	High‐fat meal	2.1 (2.0‐3.1)	11,304 (8.8)	62,219 (18.6)	59,875 (22.5)^a^	N/A	11,340(1036)	63,055 (11,282)	60,876 (13,651)^a^	136.4 (44.1)	N/A
n = 6			Fasted	1.5 (0.6‐3.0)	12,686 (17.7)	67,332 (27.7)	68,066 (27.9)		12,850 (2253)	69,420 (18,692)	70,212 (19,030)	79.3 (20.9)	
Fed versus fasted: ratio of geometric LS means (90% CI)					93.2 (75.1‐115.7)	99.5 (90.1‐109.9)	104.3 (84.9‐128.1)						
Study 2													
n = 26	50	Tablet	Fasted	0.8 (0.5‐3.0)	1280 (23.3)	4070 (24.4)	4160 (24.8)	1.1 (11.3)	1310 (305)	4190 (1110)	4290 (1140)	24.2 (5.0)	1.1 (0.1)
n = 25		Capsule		1.5 (0.5‐4.0)	1070 (24.5)	3870 (28.2)	3970 (28.6)		1100 (282)	4020 (1130)	4120 (1160)	23.9 (4.1)	
Tablet versus capsule: ratio of geometric LS means (90% CI)					119 (112‐127)	105 (101‐109)	105 (101‐109)						
Study 3													
n = 31	100	Tablet	High‐fat breakfast	3.0 (0.3‐6.0)	1350 (33.9)	8560 (28.2)	8790 (26.4)^b^	1.0 (14.4)^d^	1430 (566)	8880 (2530)	9080 (2370)	51.1 (11.8)	1.0 (0.1)
n = 31			Fasted	0.8 (0.3‐3.0)	2320 (29.4)	8750 (26.8)	8770 (25.8)^c^		2420 (688)	9060 (2530)	9060 (2460)	53.6 (9.6)	
Fed versus fasted: ratio of geometric LS means (90% CI)					0.6 (0.5‐0.7)	1.0 (0.9‐1.0)	1.0 (0.9‐1.1)						
Study 4													
n = 26	100	100‐mg tablet	Fasted	0.5 (0.3‐3.0)	2640 (26.5)	9000	9250	N/A	2720 (650)	9210 (1910)	9470 (1960)	59.7 (7.8)	N/A
n = 25		2 × 50‐mg tablets		0.5 (0.5‐3.1)	2420 (31.4)	8670	8770		2520 (692)	8870 (1860)	8990 (2000)	58.8 (7.5)	
1 × 100‐mg tablet versus 2 × 50‐mg tablets: ratio of geometric LS means (90% CI)					110.5 (102.8‐118.8)	102.7 (99.4‐106.1)	103.6 (99.5‐107.9)						

AUC_0‐t_, area under the plasma concentration‐time curve from time 0 to the time of the last quantifiable concentration, calculated using the linear trapezoidal rule; AUC_0‐_ **_∞_**, area under the plasma concentration‐time curve from time 0 extrapolated to infinity; CI, confidence interval; C_max_, maximum plasma concentration; CV, coefficient of variation; F_rel_, relative bioavailability, calculated as AUC_0_ **_‐∞_** [test]/AUC_0‐_ **_∞_** [reference]; LS, least squares; N/A, not available; SD, standard deviation; t_½_, half‐life; t_max_, time to maximum plasma concentration.

a n = 3. The pyruvate kinase parameters AUC_0‐∞_ and F_rel_ were not calculated for some subjects, as the concentration‐time profile did not exhibit a terminal log‐linear phase.

b N = 27.

c Number of observations (n) = 28.

d N = 26.

**Table 3:** Plasma Pharmacokinetic Parameters of Mitapivat for Study 5 by Dose, Formulation, and Food Status

					Geometric mean (geometric CV%)				Arithmetic mean (SD)				
Study, N	Dose (mg)	Mitapivat formulation	Fed/fasted status	t_max_ (hour), median (range)	C_max_ (ng/mL)	AUC_0‐t,_ ng•h/mL	AUC_0‐∞_ (ng•h/mL)	F_rel,_ AUC_0‐∞_ ^a^	C_max_ (ng/m)^a^	AUC_0‐t_ (ng•h/mL)	AUC_0‐∞_ (ng•h/mL)	t_½_ (hour)	F_rel_ AUC_0‐∞_
Study 5													
N = 31	50	Tablet	Fasted	0.8 (0.3‐2.0)	1010 (25.3)	3810 (31.9)	3900 (32.2)	—	1040 (254)	4010 (1470)	4110 (1520)	21.9 (6.8)	—
n = 30	50	Coated granules	Fasted	0.8 (0.5‐1.5)	1060 (27.0)	3710 (33.2)	3850 (31.5)	1.0 (11.9)	1100 (280)	3910 (1380)	4040 (1370)	22.5 (8.1)	1.0 (0.1)
n = 31	50	Coated granules	Strawberry yogurt	0.5 (0.5‐1.0)	1010 (25.5)	3810 (32.7)	3870 (33.1)	1.0 (13.7)	1040 (267)	4030 (1610)	4100 (1650)	19.5 (7.9)	1.0 (0.1)
n = 29	50	Coated granules	Chocolate pudding	1.0 (0.5‐3.0)	882 (26.2)	3870 (31.6)	4020 (29.9)	1.0 (11.5)	911 (237)	4070 (1450)	4200 (1450)	20.5 (5.8)	1.0 (0.1)
Strawberry yogurt versus fasted (coated granules): ratio of geometric LS means (90% CI)				−0.1^b^ (−0.3 to −0.1) ***p* = .0017**	97.2 (92.2‐102.0)	103.5 (99.0‐108.3)	102.0 (97.7‐106.4)						
Chocolate pudding versus fasted (coated granules): ratio of geometric LS means (90% CI)				0.3^b^ (0.1‐0.6) ***p* = .0073**	83.5 (79.1‐88.2)	102.9 (99.2‐106.6)	102.8 (99.0‐106.7)						

AUC_0‐t_, area under the plasma concentration–time curve from time 0 to the time of the last quantifiable concentration, calculated using the linear trapezoidal rule; AUC_0‐_ **_∞_**, area under the plasma concentration‐time curve from time 0 extrapolated to infinity; CI, confidence interval; C_max_, maximum plasma concentration; CV, coefficient of variation; F_rel_, relative bioavailability, calculated as AUC_0_ **_‐∞_** [fed]/AUC_0‐_ **_∞_** [fasted]; LS, least squares; SD, standard deviation; t_½_, half‐life; t_max_, time to maximum plasma concentration.

a F_rel_ was calculated for the coated‐granule formulation in the fasted condition (test) versus tablet formulation in the fasted condition (reference), and for the coated‐granule formulation with strawberry yogurt (test) or chocolate pudding (test) versus the coated‐granule formulation in the fasted condition (reference).

b The N, median, Hodges‐Lehman estimate of median difference (90% CI), and *P* value from the Wilcoxon signed‐rank test are presented.

##### Statistical Analyses

An analysis of variance was performed on the plasma mitapivat natural log‐transformed exposure parameters (using geometric LS means) for fasted subjects versus fed subjects (summarized in Table 2; arithmetic mean and standard deviation [SD] data are also presented). Due to the small sample size, for the statistical analyses the ratios of LS means were considered along with the 90% CIs of exposure LS means for fed versus fasted subjects, which were all within the 80%‐125% range. Together, the AUC and C_max_ data for mitapivat in this study suggest that there was no food effect on the bioavailability of orally administered mitapivat when administered using this capsule formulation.

#### Study 2

A total of 26 subjects (14 men, 12 women) were randomized in this study, and all subjects (100%) received a single oral dose of mitapivat 50 mg in tablet form (1 × 50 mg) and 25 of 26 subjects (96.2%) received a single oral dose of mitapivat 50 mg in capsule form (2 × 25 mg), with 1 subject withdrawn due to an adverse event (AE). The overall mean age was 35 years, and the mean BMI was 25.6 kg/m^2^.

Mean plasma concentration‐time profiles of mitapivat following single oral doses as tablet or capsule formulations are presented in Figure 1B. Following a single 50‐mg dose of mitapivat as a 1 × 50 mg tablet and as 2 × 25 mg capsules, the plasma concentration versus time profiles were characterized by a rapid absorption phase, with a geometric mean C_max_ approximately 19% higher for the tablet formulation than for the capsule formulation.

In this relative bioavailability study, the exposures of the tablet and capsule formulations (defined by AUC_0‐t,_ AUC_0‐∞_, and C_max_) were found to be similar (Table 2). However, for the tablet formulation, the t_max_ occurred earlier than for the capsule formulation, with observed tablet and capsule median t_max_ values of 0.75 and 1.50 hours, respectively (Table 2).

##### Statistical Analyses

For the AUC_0‐t_ and AUC_0‐∞_, the geometric LS mean ratios of the tablet were approximately 5% greater than those of the capsule, with lower and upper 90% CIs of 101% and 109%, respectively (Table 2). For the C_max_, the geometric LS means ratio of the tablet was approximately 19% greater than that of the capsule, with lower and upper 90% CIs of 112% and 127%, respectively (Table 2). Between‐subject variability for the AUC_0‐t_, AUC_0‐∞,_ and C_max_ was within the range of 23.3%‐28.6%. Arithmetic mean and SD data are available in Table 2.

#### Study 3

A total of 31 subjects (18 men, 13 women) were enrolled in and completed the study, and all were included in the pharmacokinetic analysis. The overall mean age was 36.5 years, and the mean BMI was 26.0 kg/m^2^.

Following oral administration of 100 mg of mitapivat (2 × 50 mg tablets), mean plasma concentration versus time profiles under fasted conditions and fed conditions were comparable (Figure 1C; Table 2). The mean peak concentration was lower, and the time to reach peak concentration was longer under fed conditions than under fasted conditions. During the distribution phase, the plasma concentrations under fed conditions were slightly higher than those under fasted conditions, but plasma concentrations in the terminal phase were similar and overlapped under both conditions (Figure 1C).

Pharmacokinetic parameters are summarized in Table 2. The total drug exposure (AUC_0‐t_ and AUC_0‐∞_) of mitapivat was similar under fasted and fed (high‐fat, high‐calorie meal) conditions. However, the peak exposure (C_max_) of mitapivat decreased by 42% under high‐fat, high‐calorie meal conditions compared with fasted conditions. In addition, the t_max_ of mitapivat was delayed under fed (high‐fat, high‐calorie meal) conditions versus fasted conditions, ranging from 0.25 to 6.03 hours and from 0.25 to 3.00 hours for fed and fasted conditions, respectively. The mean F_rel_ ratio (AUC_0‐∞_ [fed]/AUC_0‐∞_ [fasted]) was 1.00, suggesting that mitapivat's bioavailability was not affected when given with a high‐fat, high‐calorie meal.

##### Statistical Analyses

The ratio of geometric LS means of AUC_0‐t_ and AUC_0‐∞_ under fasted versus fed conditions were estimated to be 0.98 and 1.00, respectively (Table 2). The effect of a high‐fat, high‐calorie meal on exposure (AUC) of mitapivat was not statistically significant, as the 90% CIs were contained within the interval of 80%‐125% (Table 2). Conversely, the 90% CI of the C_max_ was not contained within this interval, indicating a statistically significant effect of a high‐fat, high‐calorie meal on the C_max_ of mitapivat. The t_max_ was delayed under fed compared with fasted conditions, with a median difference of 1.88 hours (*P* <.0001).

#### Study 4

A total of 26 subjects (16 men, 10 women) were enrolled, and 25 completed the study; 1 subject had an AE that led to study discontinuation. The overall mean age was 34.9 years, and the mean BMI was 26.1 kg/m^2^. Following single oral administration, mitapivat plasma concentrations over the time range from 0 to 120 hours were comparable between the 2 mitapivat formulations: 1 × 100‐mg tablet and 2 × 50‐mg tablets. Mitapivat absorption was rapid with both formulations, with mean plasma levels reaching maximum concentrations within the first hour following administration and appeared to be eliminated in a biphasic manner (Figure 1D).

Pharmacokinetic parameters are summarized in Table 2. The peak exposure (C_max_), total exposure (AUC_0‐t_ and AUC_0‐∞_), and median t_max_ of mitapivat were comparable for the 1 × 100‐mg tablet and 2 × 50‐mg tablets formulations.

##### Statistical Analyses

The ratios of geometric LS means of AUC_0‐t_ and AUC_0‐∞_ for 1 × 100‐mg mitapivat tablet and 2 × 50‐mg mitapivat tablets were estimated to be 102.7% and 103.6%, respectively (Table 2). The 90% CIs of these ratios were contained within the predefined interval of 80%‐125%, indicating that the total exposure (AUC) was similar between the 2 treatments. In addition, the 90% CIs for the ratio of geometric LS means for the C_max_ were also within the predefined limit (110.5% [90% CI, 102.83‐118.76]) indicating that the C_max_ was similar between treatments (Table 2). Arithmetic mean and SD data are also available in Table 2.

#### Study 5

A total of 32 subjects (29 men, 3 women) were enrolled in the study. Three subjects were withdrawn (1 due to an AE and 2 due to personal choice/family reasons), with 29 subjects completing the study. The overall mean age was 39.9 years, and the mean BMI was 27.7 kg/m^2^.

**Figure 2:**
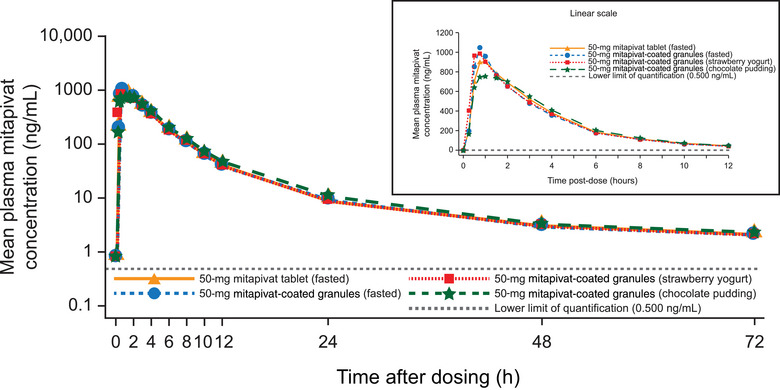
Mean plasma concentration‐time profiles following a single oral dose of 50 mg of mitapivat (tablet and coated granules) under fasted and fed (soft foods) conditions (Study 5).

Mitapivat was rapidly absorbed following administration of the tablet and coated granule formulations and also under fasted and fed conditions (Figure 2). Pharmacokinetic parameters are summarized in Table 3. Bioequivalence was established between the mitapivat tablet and the coated granule formulation when administered in a fasted state. No significant differences in the C_max_, AUC_0‐t_, or AUC_0‐∞_ were observed between these 2 formulations. In addition, a single oral administration of 50‐mg mitapivat in the coated granule formulation administered with strawberry yogurt or chocolate pudding was bioequivalent to mitapivat‐coated granules administered in a fasted state, as determined by the AUC_0‐t_ and AUC_0‐∞_ (Table 3). A slight reduction in the C_max_ was found when 50 mg of mitapivat was administered with the chocolate pudding compared with the mitapivat‐coated granules administered in a fasted state (Table 3).

##### Statistical Analyses

There were no statistically significant differences in the C_max_, AUC_0‐t_, or AUC_0‐∞_ between the fed (strawberry yogurt) and fasted states following administration of mitapivat‐coated granules (90% CIs contained within the interval of 80%‐125%). The median t_max_ occurred 0.14 hours (approximately 8 minutes) earlier in the fed (strawberry yogurt) state than in the fasted state. This difference was statistically significant (*P* = .0017) but was not considered clinically significant (Table 3). Similarly, there were no statistically significant differences reported for the comparisons of pharmacokinetic measures between mitapivat‐coated granules administered with chocolate pudding and mitapivat‐coated granules given in the fasted condition (Table 3). The t_max_ occurred 0.27 hours (approximately 16 minutes) later in the fed (chocolate pudding) state than in the fasted state (*P* = .0073), although this was not considered to be clinically significant.

### Safety

Mitapivat was found to have an acceptable safety profile and was well tolerated under both fasted and fed conditions (in Studies 1, 3, 4, and 5) and when administered as tablet and capsule formulations in adult subjects (Study 2). In Study 1, during the fed portion of the study (in which subjects were treated with doses of 700 mg of mitapivat), 3 of the 5 subjects (60%) experienced events (all were Grade 1 or 2), with 1 Grade 1 event (abdominal discomfort) considered by the investigator to be related to mitapivat; there were no Grade 3 or worse AEs, serious AEs, or deaths. One subject, who was scheduled to receive placebo during the fed period, discontinued the study due to a treatment‐related AE of anemia during the washout after the fasted period. During Study 2, 1 mild‐severity (Grade 1) AE of lice infestation (not related to the study drug) was reported in a subject receiving the mitapivat tablet formulation. This AE led to the subject discontinuing the study early; no other AEs were reported. In Study 3, 11 of the 31 subjects (35.5%) experienced at least 1 AE after administration of mitapivat or placebo (fasted and fed combined), all of which were Grade 1 or 2 in severity and had resolved by the end of the study; there were no serious AEs or AEs that led to discontinuation. In Study 4, 3 of the 26 subjects (11.5%) experienced 5 AEs after receiving the 1 × 100‐mg mitapivat tablet formulation, and 4 of the 25 subjects (16.0%) experienced 6 AEs after receiving the 2 × 50‐mg mitapivat tablet formulation. All AEs were of mild severity and had resolved by the end of the study. One event of ventricular extrasystoles was reported in a subject with a history of ventricular extrasystoles after receiving the 1 × 100‐mg mitapivat tablet formulation, and this led to discontinuation. During Study 5, 10 of the 32 subjects (31.3%) experienced at least 1 AE, the majority of which were of mild severity. The number of AEs was similar across treatment groups. One subject (receiving mitapivat as 50‐mg coated granules in strawberry yogurt on Day 1 of Period 1 and a tablet under fasted conditions on Day 1 of Period 2) had a moderate (Grade 2) AE of syncope on Day 7 of Period 2, which was considered to be unrelated to treatment, and was discontinued from the study. Other than 1 event of alanine aminotransferase increasing in 1 subject (4 days after receiving mitapivat as 50‐mg coated granules in strawberry yogurt), all AEs were resolved by the end of the study.

## Discussion

The clinical development of mitapivat represents a milestone in the treatment of PK deficiency, and the drug is now used clinically to treat hemolytic anemia in adult patients with PK deficiency.^11^, ^12^, ^16^, ^17^, ^18^, ^19^ In addition, mitapivat treatment is currently under investigation in 2 Phase 3 studies in adult patients with thalassemia, in a Phase 2/3 study in sickle cell disease, and in 2 Phase 3 studies in pediatric patients with PK deficiency (please refer to Table S3 for details of national clinical trial [NCT] references).

The Phase 1 study that included an exploratory food effect assessment (Study 1) was the first to confirm no apparent effect of food on the relative bioavailability of orally administered mitapivat in the capsule formulation (700‐mg dose in fasted versus fed [high‐fat meal] conditions). Based on these results, mitapivat was allowed to be taken with or without food in subsequent studies in adult subjects with hemolytic anemias.

A hand‐filled capsule formulation of mitapivat had been used for all clinical studies prior to Study 2. Data from this study showed that the exposure of mitapivat, defined as the AUC and C_max_, was similar following the administration of both the tablet and the capsule formulations. Consequently, the tablet formulation of mitapivat was selected for further evaluation in adult PK deficiency clinical trials. A tablet formulation was introduced in the pivotal Phase 3 studies ACTIVATE^11^ and ACTIVATE‐T,^12^ with mitapivat doses (5, 20, and 50 mg twice daily) selected on the basis of the Phase 2 DRIVE‐PK study,^6^ which used the capsule formulation. This relative bioavailability study supported using the same doses in both the tablet formulation and the capsule formulation, with no dose adjustment required prior to their use in the pivotal studies.

Subsequently, a study on food effect for the mitapivat tablet formulation was initiated. In a Phase 2 study that was ongoing in subjects with thalassemia (core study period completed) and in the planned (currently ongoing) pivotal Phase 3 studies for patients with thalassemia, a higher dose of mitapivat (100 mg twice daily) was considered (please refer to Table S3 for NCT reference). To avoid study repetition and assessment of the food effect with multiple doses of mitapivat, the effect of food on a single 100‐mg dose of mitapivat in the tablet formulation was investigated in the Phase 1 study in healthy adult subjects under fasted and fed (high‐fat, high‐calorie meal) conditions. This study found that, despite a decreased C_max_ of mitapivat under fed (high‐fat, high‐calorie meal) conditions, the total drug exposure of mitapivat was similar under fasted and fed states. Statistical analysis (90% CIs) also indicated that the effect of high‐fat, high‐calorie meals on the exposure (AUC) of mitapivat was not statistically significant (Table 2). These study findings supported the use of mitapivat in adult subjects with PK deficiency and provided the rationale for the administration of mitapivat with or without food in the Phase 3 thalassemia studies.

A bridging study to characterize and compare the pharmacokinetics and assess the safety of the 100‐mg tablet formulation and the 50‐mg tablet formulation in healthy adult subjects further supported the formulation development of mitapivat. Despite the availability of in vitro dissolution data, an additional Phase 1 clinical study (please refer to Table S3 for NCT reference) was warranted to compare the bioavailability between 1 × 100‐mg and 2 × 50‐mg mitapivat tablets, as the 100‐mg tablet formulation was planned for use in Phase 3 trials of thalassemia. This study found that the total drug exposure of mitapivat was comparable between the 2 tablet formulations (1 × 100 mg and 2 × 50 mg). These data and the results of a prior Phase 2 trial in adult patients with nontransfusion‐dependent α‐ or β‐thalassemia, which reported tolerable safety and efficacy of mitapivat with twice‐daily dosing,^13^ provided the rationale to examine the 1 × 100‐mg tablet twice‐daily dose in the ongoing pivotal Phase 3 thalassemia trials ENERGIZE and ENERGIZE‐T (please refer to Table S3 for NCT reference).

As part of mitapivat's pediatric clinical development, Study 5 was conducted to evaluate and compare a pediatric coated‐granule formulation of mitapivat with the adult formulation, as well as to evaluate the impact of 2 soft foods suitable for children on the bioavailability of the pediatric coated‐granule formulation. The mitapivat drug substance is highly soluble between a pH of 1.1 and 5.5, with solubilities being 9.72, 3.20, 2.23, and 0.34 mg/mL at a pH of 1.1, 2.8, 4.8, and 5.5, respectively.^15^ The solubility, however, reduces below the solubility limit of 0.20 mg/mL at a pH of 6.0 to 8.0.^15^ Therefore, strawberry yogurt (approximate pH range, 4.0‐5.0) and chocolate pudding (approximate pH range, 5.5‐6.5) were selected for evaluation of mitapivat pharmacokinetics in soft foods across a wide pH range. Prior to the commencement of this study, in vitro and palatability studies with various pediatric foods were conducted. The palatability studies indicated that soft foods, including chocolate pudding, strawberry yogurt, and sweetened/unsweetened apple sauce, would be appropriate for assessment in the mitapivat pediatric trials, as these were the most palatable for children and the easiest to consume. In Study 5, the potential enhancement or delay of mitapivat absorption with the soft foods considered was deemed minimal and neither was considered clinically significant by investigators. When compared with the fasted group, there were no statistically significant differences in pharmacokinetic parameters (AUC_0‐t_ and AUC_0‐∞_) when mitapivat‐coated granules were administered with soft foods (strawberry yogurt and chocolate pudding). These important findings informed mitapivat dosing instructions for the 2 ongoing pediatric PK deficiency clinical studies: ACTIVATE‐Kids and ACTIVATE‐KidsT (please refer to Table S3 for NCT reference).

In all of these trials, the relative bioavailability was assessed for the whole tablet or capsule formulation of mitapivat. No evaluations have been conducted to date to assess the effect of food on the relative bioavailability of mitapivat in a chewed or crushed tablet form.

To summarize, data from the 5 Phase 1 studies conducted in healthy adult subjects suggested that the pharmacokinetics of mitapivat were similar across different formulations. Additionally, there was no clinically significant food effect on the relative bioavailability of orally administered mitapivat. Finally, mitapivat demonstrated an acceptable safety profile and was well tolerated during all Phase 1 studies.

## Conclusion

During the Phase 1 trials evaluating multiple formulations of mitapivat in healthy adults, the plasma total exposure of mitapivat was found to be similar in the fed (high‐fat meal) and fasted states after oral administration of the capsule and tablet formulations. Furthermore, mitapivat was associated with an acceptable safety profile and was well tolerated under fasted and fed conditions, irrespective of formulation, with most events reported to be mild or moderate in severity. These data continue to support the use of mitapivat treatment for hemolytic anemias and may help inform health care professionals on dosing administration recommendations of mitapivat in practice. The administration instructions for mitapivat with regard to food state that patients may take mitapivat tablets with or without food.

## Conflicts of Interest

Varsha Iyer and Karen Sullivan are former employees and shareholders of Agios Pharmaceuticals, Inc. Yan Yan and Peter Hawkins are employees and shareholders of Agios Pharmaceuticals, Inc.

## Funding

This study was funded by Agios Pharmaceuticals, Inc.

## Supporting information



Supporting Information

## Data Availability

Qualified researchers may request access to related clinical study documents. Data‐sharing requests may be sent to datasharing@agios.com.
