# Supplementary material for: Relative Bioavailability Studies With Mitapivat: Formulation and Food Effect Assessments in Healthy Subjects
Source: Clin Pharmacol Drug Dev. 2024 Oct 25;13(12):1271–82. doi: 10.1002/cpdd.1481 (PMC11609058; doi:10.1002/cpdd.1481)
Supplement: Supplementary file 1 — Supporting Information [file CPDD-13-1271-s001.docx]

**Supplementary Information**

**Figure S1.**


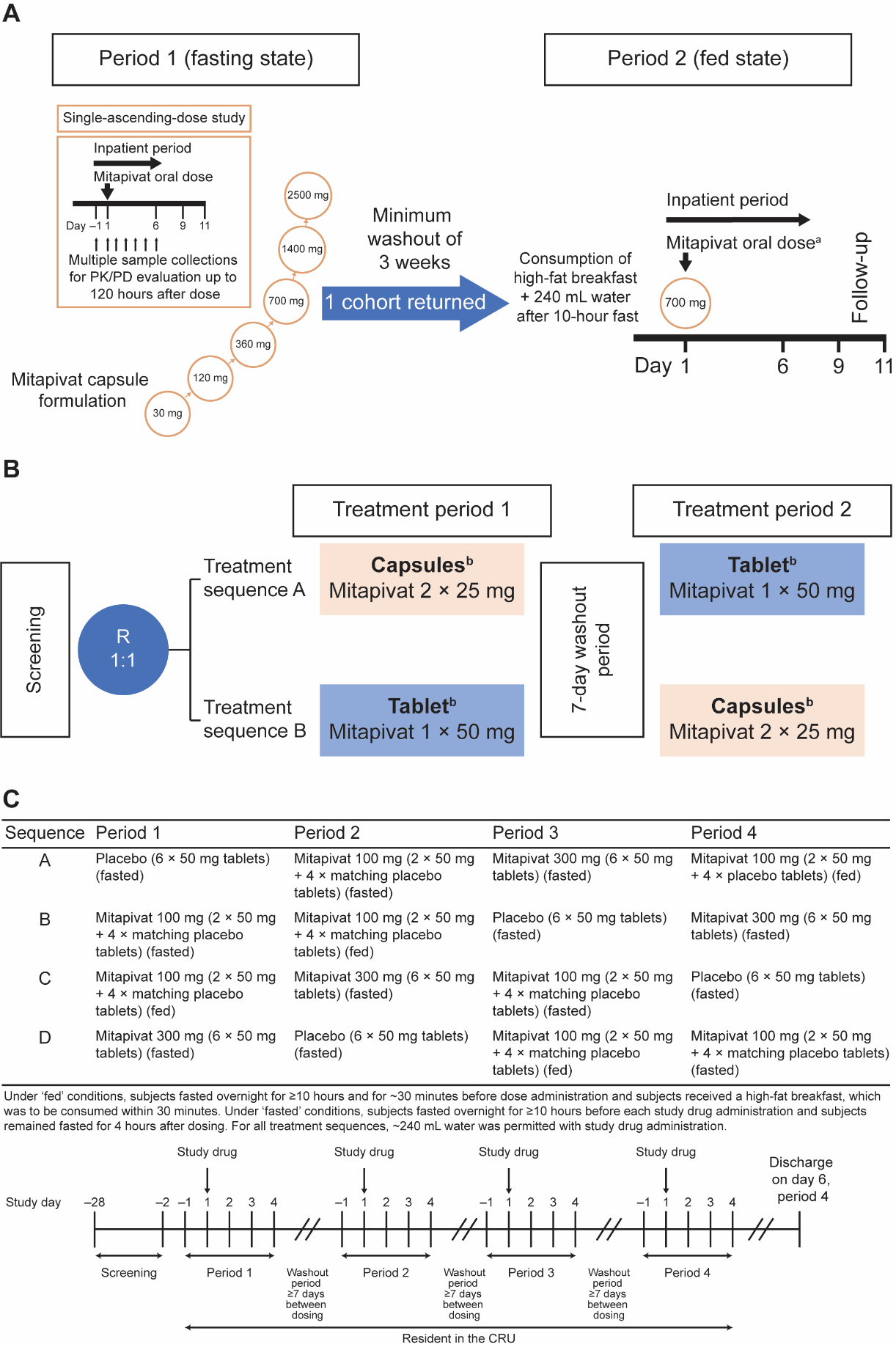


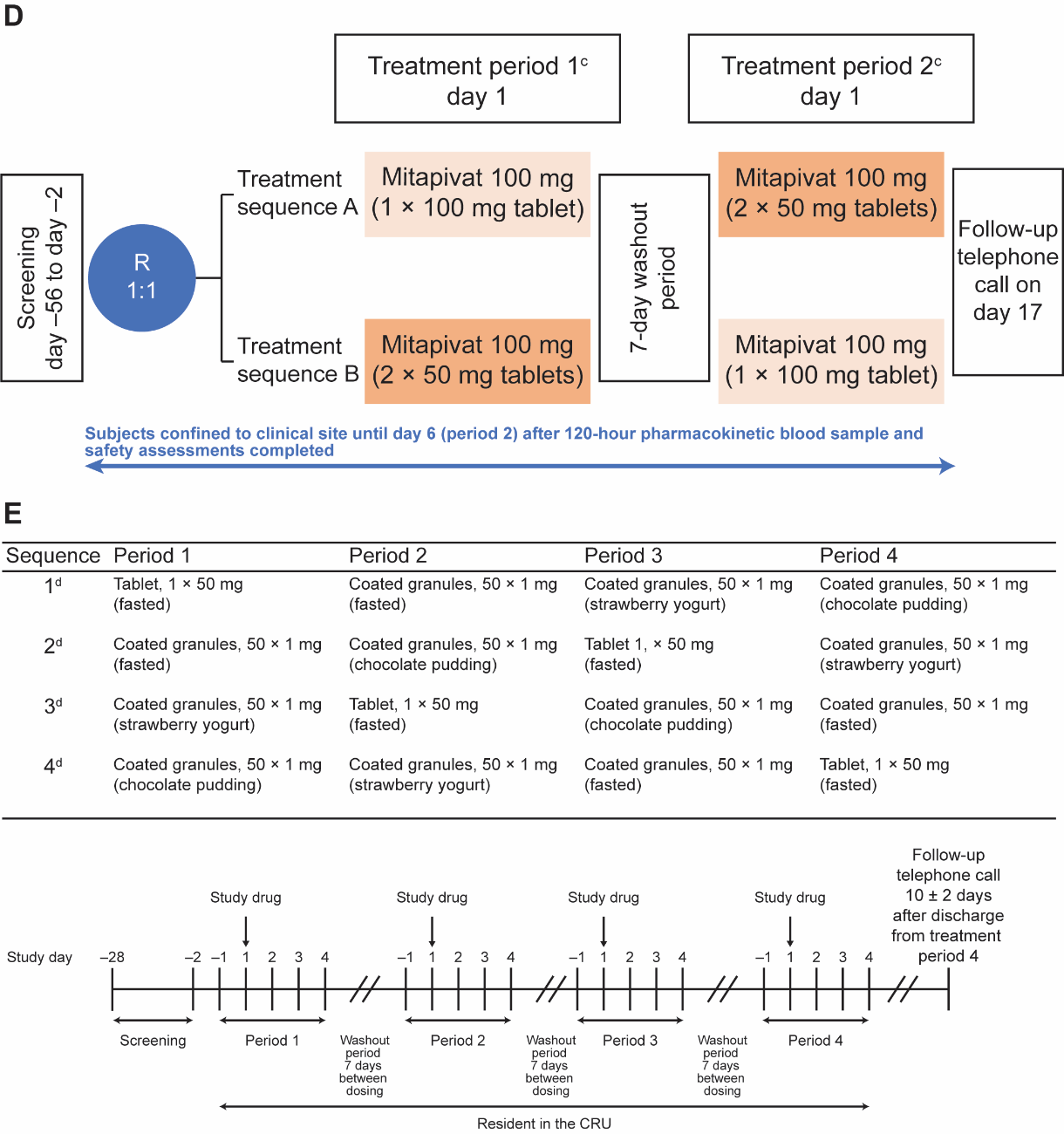


**Figure S1.** Mitapivat study designs in healthy subjects. (A) Study 1: mitapivat was administered in capsule formulation as a single oral dose in healthy adult males and females of non-childbearing potential. Eight subjects were enrolled into six sequential dose cohorts. Within each dose cohort, subjects were randomized to receive mitapivat (N = 6) or placebo (N = 2) based on a block randomization scheme. The safety and tolerability of the mitapivat doses (30, 120, 360, 700, 1400, and 2500 mg) were assessed under fasted conditions (period 1). The mitapivat 700 mg cohort returned for subsequent dosing under fed conditions to assess the effect of food on the bioavailability of mitapivat (period 2). (B) Study 2: this study compared the relative bioavailability and safety of the mitapivat tablet and capsule formulations after single-dose administration in healthy adults. On day 1, all eligible subjects were randomized 1:1 to one of two treatment sequences, with subjects receiving two single oral doses of mitapivat 50 mg, one as a tablet formulation and the other as a capsule formulation, separated by a washout period of 7 days. All doses were administered with approximately 240 mL of water and were preceded by a fast of at least 10 hours and followed by a fast of at least 2 hours. Plasma samples for pharmacokinetic analysis were collected before the dose of mitapivat and at time intervals up to 72 hours post-dose. (C) Study 3: this study assessed the effect of a high-fat (with fat representing approximately 50% of the total caloric content of the meal) and high-calorie (approximately 800–1000 calories) meal on a single dose of mitapivat 100 mg. Subjects received a single oral dose of mitapivat 100 mg (administered as a tablet formulation) or placebo on the first day of each period for a total of four doses, with a washout period of at least 7 days between each dose. Only the mitapivat treatment sequences (sequences B, C, and D) were considered for the purpose of this study. (D) Study 4: this study compared the pharmacokinetics and safety of mitapivat 100 mg tablet formulation with mitapivat 2 × 50 mg tablet formulation after a single-dose administration of mitapivat 100 mg under fasted conditions in healthy adult subjects. On day 1 of each treatment period, after an overnight 10-hour fast, subjects received either a single oral dose of mitapivat 100 mg (1 × 100 mg tablet formulation) or a single oral dose of mitapivat 100 mg (2 × 50 mg tablet formulation). In each treatment period, subjects remained fasted for 4 hours after dosing. Water was permitted as desired except for 1 hour before and 1 hour after administration of the study drug, with the exception of water needed for study drug administration (approximately 240 mL). In each treatment period, serial blood samples for the pharmacokinetic analysis of mitapivat were collected before dosing and up to 120 hours after dosing. (E) Study 5: the relative bioavailability of the mitapivat coated granule formulation (pediatric formulation) was compared with that of the tablet (adult formulation) following administration of a single oral dose of mitapivat 50 mg under fasted conditions in healthy adult subjects. Eight subjects were randomly assigned to one of four treatment sequences. Each sequence consisted of four periods during which each subject received one of the four treatment regimens listed in the table. For all treatment sequences, water (approximately 240 mL) was permitted to be taken with study drug administration. A washout period of 7 days ensured elimination of the study drug prior to dosing in the subsequent periods.

^a^Followed by a 4-hour fast.

^b^Taken with 250 mL of water while subject was in the fasted state (i.e., having fasted for ≥10 hours pre-dose and ≥2 hours post-dose).

^c^In each treatment period, mitapivat was administered as a single oral dose after an overnight fast of ≥10 hours, with subjects remaining in the fasted state for 4 hours after dosing. Water was permitted as desired except for 1 hour before and 1 hour after mitapivat administration, with the exception of water needed for study drug administration (~240 mL).

^d^For all treatment sequences, ~240 mL of water was permitted with study drug administration.

CRU, clinical research unit; PK/PD, pharmacokinetic/pharmacodynamic; R, randomization.

**Table S1.** IRBs and Study Site Locations for the Five Phase I Trials

| **Study identifier** | **Study description** | **IRB and location** | **Study site and location** |
| --- | --- | --- | --- |
| **NCT02108106 Study 1** | Preliminary food effects with capsule formulation (single ascending dose of mitapivat) | Aspire IRB Santee, CA 92071, USA | Parexel Baltimore EPCU  Baltimore, MD 21225, USA |
| **NCT03397329 Study 2** | Relative bioavailability and safety of mitapivat tablet and capsule formulations (1 × 50 mg) | Salus IRB Austin, TX 78758, USA | Covance Clinical Research Unit Dallas, TX 75247, USA |
| **NCT04472832 Study 3** | Pharmacokinetics, safety, and tolerability of mitapivat tablet formulation (1 × 100 mg) in the presence and absence of a high-fat meal | Salus IRB Austin, TX 78758, USA | PPD Development, LP Austin, TX 78744, USA |
| **NCT04696393 Study 4** | Pharmacokinetics and safety of mitapivat 100 mg tablet formulation compared with mitapivat 2 × 50 mg tablet formulation | Salus IRB Austin, TX 78758, USA | PPD Development, LP Austin, TX 78744, USA |
| **NCT04565678 Study 5** | Relative bioavailability and food effect of the pediatric coated granule formulation of mitapivat | Salus IRB Austin, TX 78758, USA | Covance Clinical Research Unit Dallas, TX 75247, USA |

IRB, institutional review board.

**Table S2.** Summary of Inclusion and Exclusion Criteria across the Five Phase 1 Studies

| **Study** | **Inclusion criteria** | **Exclusion criteria** |
| --- | --- | --- |
| **Study 1** | - Male or female (of non-childbearing potential) subjects aged ≥18 to ≤60 years - Male subjects must have used a condom with spermicide as contraception from day 1 until 30 days following the last dose of the study drug - Body weight ≥50 kg and BMI ≥18.5 to ≤32 kg/m^2^ - Non-smokers and must have not used other nicotine-containing products for ≥3 months before screening - Must have not used caffeine- or xanthine-containing products for 48 hours before dosing through 120 hours post-dose (day 6) - Must have not performed strenuous exercise for 72 hours before admission through final follow-up visit | - Systolic BP ≥140 mmHg or diastolic BP of ≥90 mmHg after 10 minutes of supine rest - Male subjects with QTcF interval ECG >450 msec; female subjects with QTcF interval ECG >470 msec on screening/day 1 (pre-dose) ECG - Glucose-6-phosphate-dehydrogenase deficiency - A history of any prior malignancy, except basal cell or squamous cell carcinomas of the skin, cervical carcinoma in situ, or other malignancies curatively treated and with no evidence of disease for ≥1 year - A pre-existing condition that interferes with normal gastrointestinal anatomy or motility, and/or hepatic and/or renal function that could interfere with the absorption, metabolism, and/or excretion of the study drug (cholecystectomy was not exclusionary) |
| **Study 2** | - Male or female subjects aged ≥18 to ≤55 years - Female subjects who were not pregnant or breastfeeding and either were of non-childbearing potential or agreed to abstain from sexual intercourse or use highly effective contraceptive methods - Male subjects must have used highly effective contraceptive methods from day 1 until 90 days following the last dose of the study drug - Body weight ≥50 kg and BMI ≥18.5 to ≤29.0 kg/m^2^ - Non-smokers and must have not used other nicotine-containing products for ≥12 months before screening | - Systolic BP ≥140 mmHg (≥150 mmHg in subjects >45 years of age) or diastolic BP of ≥90 mmHg after 5 minutes of supine rest - Glucose-6-phosphate-dehydrogenase deficiency - A history of any prior malignancy, except those curatively treated and with no evidence of disease for ≥1 year - A heart rate-corrected QT interval by QTcF of >450 msec on screening ECG |
| **Study 3** | - Male or female subjects aged ≥18 to ≤55 years - Female subjects who were not pregnant or breastfeeding and either were of non-childbearing potential or agreed to abstain from sexual intercourse or use highly effective contraceptive methods - Male subjects must have used highly effective contraceptive methods from day 1 until 90 days following the last dose of the study drug - BMI ≥18.0 to ≤32.0 kg/m^2^ | - QTcF >450 msec - Glucose-6-phosphate-dehydrogenase deficiency - A history of any prior malignancy, except basal cell or squamous cell carcinomas of the skin, cervical carcinoma in situ, or other malignancies curatively treated and with no evidence of disease for ≥5 years |
| **Study 4** | - Male or female subjects aged ≥18 to ≤64 years - Body weight ≥50 kg and BMI ≥18.0 to ≤32.0 kg/m^2^ - Female subjects who were not pregnant or breastfeeding and either were of non-childbearing potential or agreed to abstain from sexual intercourse or use highly effective contraceptive methods - Male subjects must have used highly effective contraceptive methods from day 1 until 90 days following the last dose of the study drug | - Clinically significant cardiac history or presence of ECG findings as determined at screening, including QTcF >450 msec - Systolic BP >150 or <90 mmHg, diastolic BP >90 or <50 mmHg, and pulse rate >100 or <45 beats per minute on two consecutive readings at screening - A history of any prior malignancy, except those curatively treated and with no evidence of disease for ≥1 year - Liver test results above the upper limit of normal at screening - eGFR <60 mL/min/1.73 m^2a^ |
| **Study 5** | - Male or female subjects aged ≥18 to ≤55 years - BMI ≥18.0 to ≤32.0 kg/m^2^ - Female subjects who were not pregnant or breastfeeding - Females of childbearing potential and males who agreed to use two methods of contraception (females, one primary highly effective and one secondary method; male, one barrier and one acceptable method of contraception) until 90 days after the follow-up telephone call - In good health as determined by medical history, physical exam, vital signs, 12-lead ECG, and clinical laboratory findings | - Significant history/clinical manifestation of metabolic, allergic, dermatologic, hepatic, renal, hematologic, pulmonary, cardiovascular, gastrointestinal, neurologic, respiratory, endocrine, or psychiatric disorder - Clinically significant history/presence of abnormal ECG findings at screening, including QTcF >450 msec - History of stomach/intestinal surgery - History of malignancy/received anticancer treatment ≤5 years before study - Intolerance/allergy to any drug compound, including the two soft foods administered (chocolate pudding and strawberry yogurt) - Liver test results above the ULN at screening |

BMI, body mass index; BP, blood pressure; ECG, electrocardiogram; eGFR, estimated glomerular filtration rate; QTcF, corrected QT interval: Fridericia’s method; ULN, upper limit of normal.

^a^Using the Chronic Kidney Disease Epidemiology Collaboration 2009 equation.

**Table S3.** NCT References for Mitapivat Studies Cited in Discussion

| **Indication** | **NCT reference** |
| --- | --- |
| **Thalassemia (ongoing)** | ClinicalTrials.gov. A study evaluating the efficacy and safety of mitapivat in participants with non-transfusion-dependent alpha- or beta-thalassemia (α- or β-NTDT) (ENERGIZE) (NCT04770753). 2023. https://clinicaltrials.gov/study/NCT04770753. Accessed June 27, 2024. |
|  | ClinicalTrials.gov. A study evaluating the efficacy and safety of mitapivat in participants with transfusion-dependent alpha- or beta-thalassemia (α- or β-TDT) (ENERGIZE-T) (NCT04770779). 2023. https://clinicaltrials.gov/study/NCT04770779. Accessed June 27, 2024. |
|  | ClinicalTrials.gov. A study to determine the efficacy, safety, pharmacokinetics, and pharmacodynamics of AG-348 in adult participants with non-transfusion-dependent thalassemia (NCT03692052). 2023. https://classic.clinicaltrials.gov/ct2/show/NCT03692052. Accessed June 27, 2024. |
| **Sickle cell disease (ongoing)** | ClinicalTrials.gov. A study evaluating the efficacy and safety of mitapivat (AG-348) in participants with sickle cell disease (RISE UP) (NCT05031780). 2023. https://clinicaltrials.gov/ct2/show/NCT05031780. Accessed June 27, 2024. |
| **PK deficiency (ongoing)** | ClinicalTrials.gov. A study to evaluate the efficacy and safety of mitapivat in pediatric participants with pyruvate kinase deficiency (PKD) who are regularly transfused, followed by a 5-year extension period (ACTIVATE-KidsT) (NCT05144256). 2023. https://clinicaltrials.gov/study/NCT05144256. Accessed June 27, 2024. |
|  | ClinicalTrials.gov. A study to evaluate the efficacy and safety of mitapivat in pediatric participants with pyruvate kinase deficiency (PKD) who are not regularly transfused, followed by a 5-year extension period (ACTIVATE-Kids) (NCT05175105). 2023. https://clinicaltrials.gov/study/NCT05175105. Accessed June 27, 2024. |
| **Healthy adults (completed)** | ClinicalTrials.gov. A study to evaluate the pharmacokinetics, safety, and tolerability of mitapivat (AG-348) in healthy adult participants (NCT04472832). 2023. https://www.clinicaltrials.gov/study/NCT04472832. Accessed June 27, 2024. |
|  | ClinicalTrials.gov. A study to compare the pharmacokinetics and safety of mitapivat 100 mg tablet formulation with mitapivat 2 × 50 mg tablet formulation in healthy adult participants (NCT04696393). 2023. https://www.clinicaltrials.gov/study/NCT04696393. Accessed June 27, 2024. |

NCT, national clinical trial; PK, pyruvate kinase.
